# Identification of 14-dehydroergosterol as a novel anti-inflammatory compound inducing tolerogenic dendritic cells

**DOI:** 10.1038/s41598-017-14446-1

**Published:** 2017-10-24

**Authors:** Yasuhisa Ano, Kumiko Ikado, Kazutoshi Shindo, Hideki Koizumi, Daisuke Fujiwara

**Affiliations:** 1 Research Laboratories for Health Science & Food Technologies, Kirin Company Ltd, 1-13-5 Fukuura Kanazawa-ku, Yokohama-shi, Kanagawa 236-0004 Japan; 2 Central Laboratories for Key Technologies, Kirin Company Ltd, 1-13-5 Fukuura Kanazawa-ku, Yokohama-shi, Kanagawa 236-0004 Japan; 3 Department of Food and Nutrition, Japan Women’s University, 2-8-1 Mejirodai, Bunkyo-ku, Tokyo 112-8681 Japan

## Abstract

Tolerogenic dendritic cells (DCs) have the ability to induce regulatory T cells and play an important role in preventing chronic inflammatory and autoimmune diseases. We have identified a novel compound, 14-dehydroergosterol, from Koji, a Japanese traditional food material fermented with fungi. 14-dehydroergosterol is an ergosterol analogue with a conjugated double bond, but the activity of 14-dehydroergosterol is much higher than that of ergosterol. 14-dehydroergosterol induces the conversion of murine bone marrow (BM)-derived DCs and differentiated DCs into tolerogenic DCs, in which the production of IL-12 is suppressed and that of IL-10 is increased. In a co-culture experiment, DCs treated with 14-dehydroergosterol induced the conversion of naïve CD4-positive T cells into regulatory T cells. In a murine model of multiple sclerosis, experimental autoimmune encephalopathy, 14-dehydroergosterol suppressed the clinical score and inflammatory responses of myeloid DCs and T cells to myelin oligodendrocyte glycoprotein. 14-dehydroergosterol-treated human DCs induced from PBMCs also showed a tolerogenic phenotype. This is the first report to identify a novel compound, 14-dehydroergosterol, that induces DCs to convert to a tolerogenic type. 14-dehydroergosterol is contained in various fermented foods based on Koji, so 14-dehydroergosterol might be a helpful aid to prevent chronic inflammatory and autoimmune diseases.

## Introduction

Dendritic cells (DCs) comprise a heterogeneous population of professional antigen-presenting cells that potently stimulate innate primary immune responses and possess the ability to regulate both innate and adaptive immunity^1–4^. DCs regulate T cell responses via the production of co-stimulatory molecules, cytokines and chemokines. These molecules are induced by the microenvironment and sensed through receptors such as TLRs and NOD-like receptors^5^. Immature DCs do not induce primary immune responses because they do not express the requisite co-stimulatory molecules, nor do they express antigenic peptides as stable complexes with major histocompatibility complex (MHC) molecules. Immature DCs effectively capture and process exogenous antigens in peripheral tissues, which lead to their maturation^6^. The maturing DCs migrate to lymphoid tissues, where they interact with naive T cells through the signals of both MHC molecules that present antigen-peptides and co-stimulatory molecules. The maturation of DCs is associated with a decrease in or absence of antigen uptake, high expression of MHC class II and accessory molecules, and the production of IL-12 upon stimulation^7–12^.

Immature or maturation-resistant DCs are more likely to promote tolerance, although phenotypically mature myeloid DCs (mDCs) and plasmacytoid DCs also demonstrate tolerogenic potential^13,14^. The proportion of resting or immature DCs to activated or mature DCs may also determine the induction of tolerance. Some tolerogenic DCs display phenotypic maturation but lack functional maturation, which is characterized by low expression of MHC and co-stimulatory molecules, low production of IL-12, and high production of IL-10^13–15^. Tolerogenic DCs have the ability to induce regulatory T cells (Tregs)^16^. Tregs are predominantly CD4-positive and CD25-positive cells, and the transcription factor fork head winged helix protein-3 (FoxP3) is specific marker for Tregs^17^. Tregs are essential for maintaining peripheral tolerance, preventing and limiting autoimmune diseases, such as type 1 diabetes and multiple sclerosis, and preventing chronic inflammatory diseases, such as asthma and inflammatory bowel disease^18,19^. Other than retinoic acids^20,21^, however, few compounds that induce Tregs, especially compounds derived from food components that are easy to consume in daily life, have been reported.

Owing to their potential health benefits, Japanese food products are receiving increasing attention worldwide; however, the health-promoting mechanisms and responsible ingredients in Japanese traditional foods have not been sufficiently elucidated. As a first step, we evaluated the ability of various food materials to induce the conversion of bone marrow (BM)-derived cells into tolerogenic DCs, thereby identifying the Japanese traditional food material Koji, which comprises a cereal crop such as rice, wheat, and soy that has been fermented with fungi. The fungi belong to the genus *Aspergillius*, which produces various metabolites and enzymes^22,23^. Koji is the ingredient of various Japanese traditional foods such as miso, soy sauce, Japanese pickle, sake, distilled spirit, and so on. Whereas there are various reports regarding the benefits of Koji on taste and flavor^24^, there are few on health benefits. In the present study, we identified 14-dehydroerogosterol (14-DHE) as a compound in Koji that induces tolerogenic DCs. 14-DHE has been previously reported as a sterol derivative^25^; to our knowledge, however, no studies have described its functions or biological activities. We have investigated the properties of 14-DHE from Koji and elucidated its functional ability in the prevention of autoimmune and chronic inflammatory disease.

## Results

### Identification of 14-DHE from wheat bran fermented with *Aspergillus* as an inducer of tolerogenic DCs

**Figure 1:**
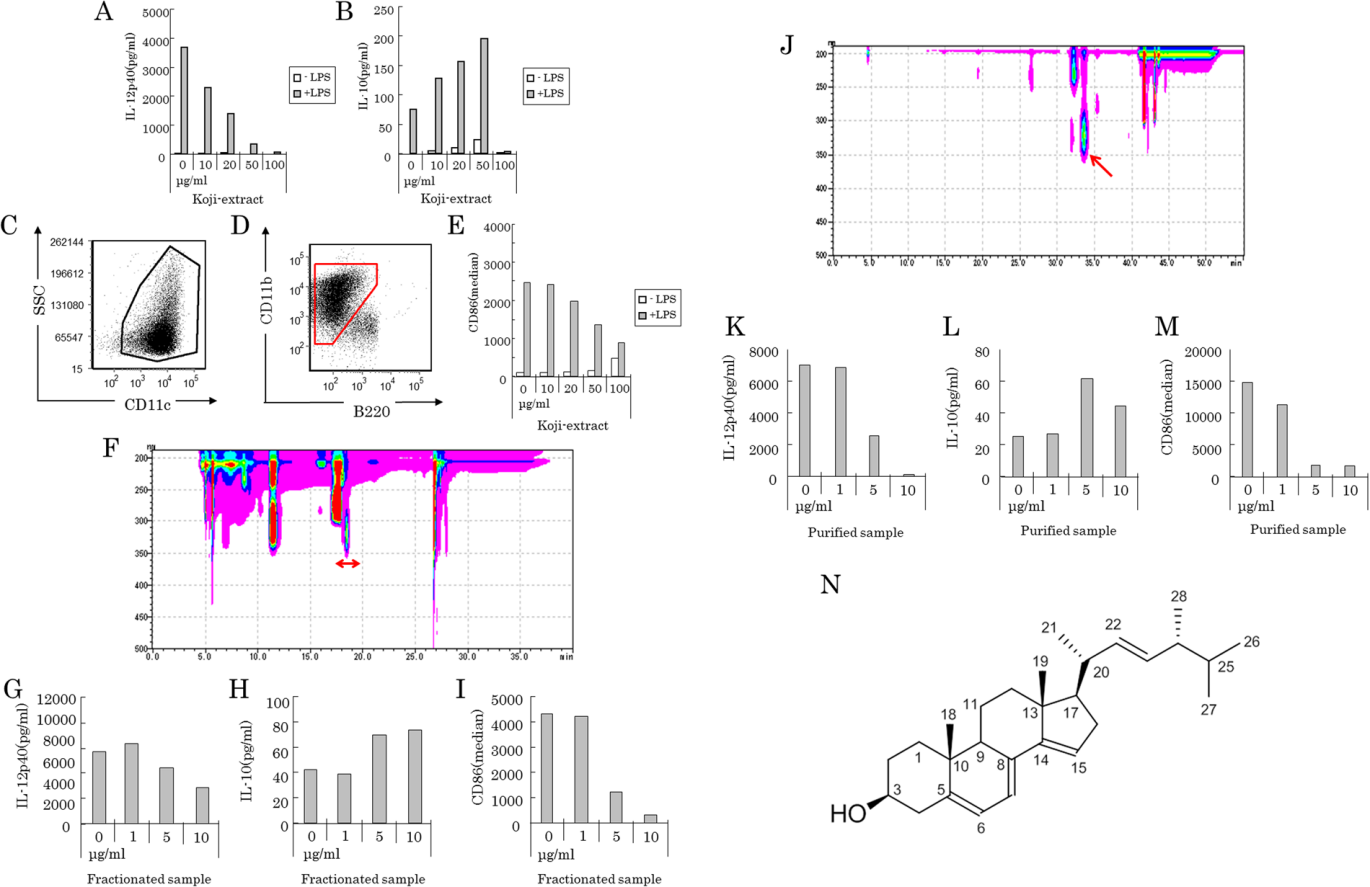
Identification of 14-DHE, a novel anti-inflammatory compound inducing tolerogenic DCs, from wheat bran fermented with fungi. Bone marrow cells were incubated in medium containing 100 ng/ml of Flt-3L and in the presence of the wheat brain extract fermented with fungi, and then stimulated by 5 ng/ml of LPS on day 7. Each sample was evaluated in the two wells. (**A** and **B**) IL-12p40 and IL-10 in the supernatant was quantified by ELISA. (**C**–**E**) Cells were gated as shown in (**C** and **D**), and the expression of CD86 was analyzed as fluorescent intensity (**E**). (**F**) HPLC of the extract of wheat brain fermented with fungi with detection by photodiode array detector (PDA). (**G**–**I**) The fraction indicated by red arrow in (**F**) displayed the highest activity in myeloid DCs including suppression of IL-12p40 production (**G**), increasing IL-10 production (**H**), and suppression of CD86 expression (**I**). (**J**) HPLC of the fraction indicated by the arrow in (**F**) with detection by PDA. (**K**–**M**) The compound indicated by the red arrow in (**J**) affected inflammatory responses in myeloid DCs including suppression of IL-12p40 production (**K**), increasing IL-10 production (**L**), and suppression of expression of CD86 (**M**) in response to LPS stimulation. (**N**) Structural analysis using 1H-NMR and identification of 14-DHE as the active compound. Each bar is means of 2 wells per sample.

As a result of searching for materials inducing tolerogenic DCs, the whole extract from wheat bran fermented with *Aspergillus awamori* was found to lead to reduced IL-12p40 production (Fig. 1A) and increased IL-10 production (Fig. 1B) in response to LPS stimulation in BM-derived mDCs. The mDCs were gated as CD11c-positive, CD11b-positive, and B220-negative cells (Fig. 1C and D) after treatment with the wheat bran extract, and showed reduced expression of CD86 in response to LPS stimulation (Fig. 1E).

To identify the compound responsible for these changes, the extract from wheat bran fermented with *Aspergillus awamori* was fractionated by HPLC, and the fraction displaying the strongest suppression of the production of IL-12p40 and the expression of I-A/I-E, CD86 and CD80 was re-fractionated. The fraction shown between the two red arrows in Fig. 1A showed the highest suppression after fractionation by an UK-silica column in a hexane-isopropanol (98:2) gradient (Fig. 1F). This fraction led to reduced IL-12p40 production (Fig. 1G) and increased IL-10 production (Fig. 1H); it also reduced the expression of CD86 on mDCs (Fig. 1I). The fraction was re-fractionated by using a C30-UG-5 column with an acetonitrile-isopropanol (99:1) gradient (Fig. 1J). The fraction indicated by the red arrow in Fig. 1J displayed the same activity as the fraction indicated in Fig. 1F (Fig. 1K, L, and M). As a result of this repeated fractionation and purification, 14-DHE was identified as the active component by MS and NMR analyses (Fig. 1N). In fermented wheat bran, 14-DHE exhibited the highest specific activity for reducing both the production of IL-12p40 and the expression of co-stimulatory molecules in mDCs in response to LPS stimulation.

### 14-DHE displayed superior induction of tolerogenic DCs as compared with ergosterol and other analogues

**Figure 2:**
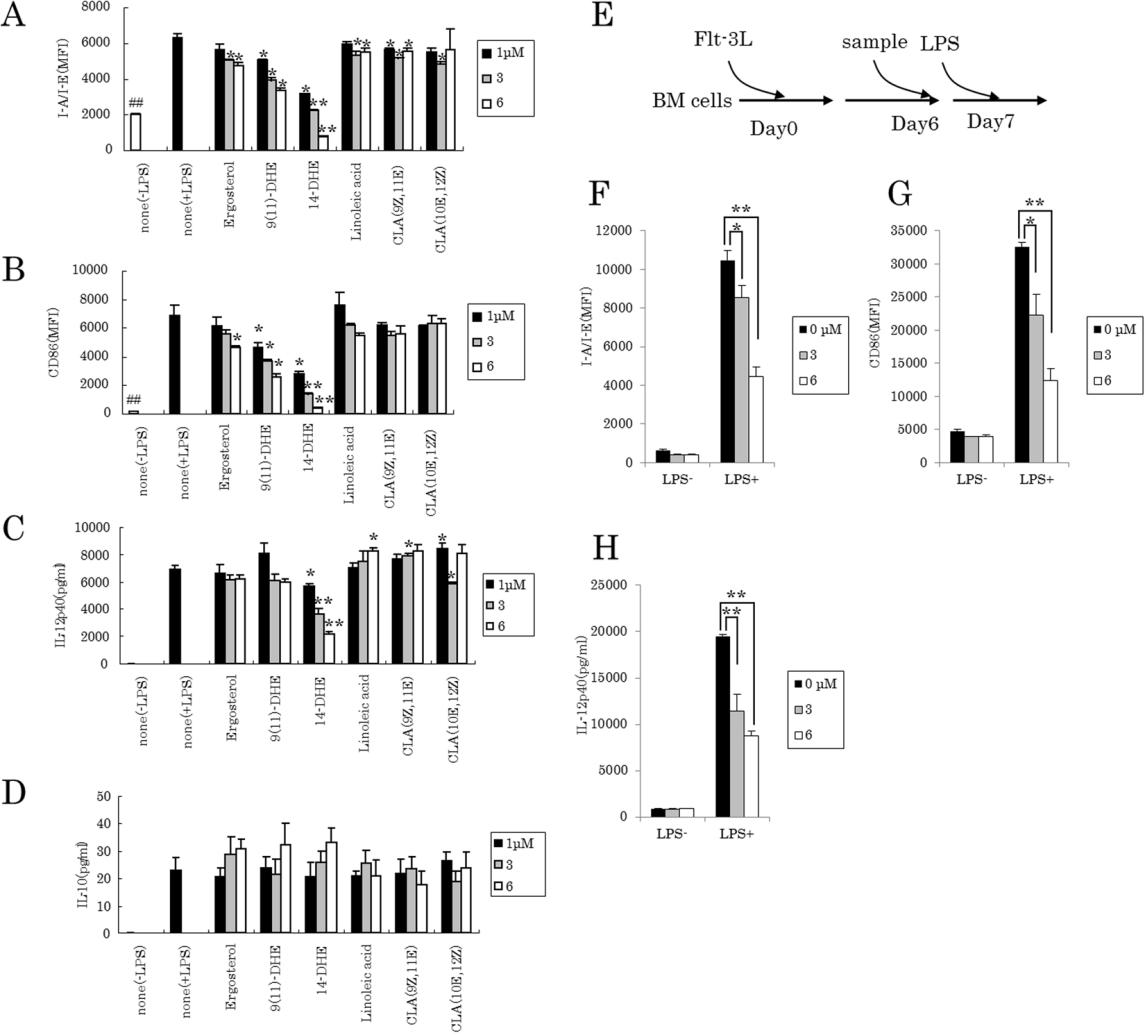
Effect of 14-DHE on DC differentiation and DC modification. (**A**–**D**) Bone marrow cells were cultured with medium containing 100 ng/ml of Flt-3L and 1, 3 or 6 μM 14-DHE, and then treated with 5 ng/ml of LPS on day 7. After LPS treatment overnight, the expression of I-A/I-E (**A**) and CD86 (**B**) on CD11b and CD11c-positive myeloid DCs was measured by flow cytometry, and the concentration of IL-12p40 (**C**) and IL-10 (**D**) in the cell supernatant was quantified by ELISA. (**E**–**H**) Bone marrow cells were cultured with medium containing 100 ng/ml of Flt-3L, treated with 1, 3 or 6 μM 14-DHE on day 6, and then with 5 ng/ml of LPS on day 7 schemed in (**E**). After LPS treatment overnight, the expression of I-A/I-E (**F**) and CD86 (**G**) on CD11b and CD11c-positive cells was analyzed by flow cytometry, and the concentration of IL-12p40 (**H**) in the culture supernatant was quantified by ELISA. Data are means ± SEM of 3 wells per sample. *p < 0.05 and **p < 0.01.

When DCs were incubated in the presence of 14-DHE, the resultant DCs showed reduced expression of I-A/I-E (Fig. 2A) and CD86 (Fig. 2B), as well as decreased production of IL-12p40 (Fig. 2C) in a concentration-dependent manner in response to LPS stimulation. At the same time, 14-DHE increased production of IL-10 (Fig. 2D). In addition, when 14-DHE was added after DC differentiation schemed in Fig. 2E, i.e., on day 6, the expression of I-A/I-E (Fig. 2F) and CD86 (Fig. 2G), and the production of IL-12p40 (Fig. 2H) in response to LPS stimulation were decreased as compared with control cells. So, 14-DHE displayed the capability to induce DCs not dependent on the level of differentiation into tolerogenic type.

14-DHE is an analogue of ergosterol and has a C-14 conjugated double bond. Therefore, its effects were compared with those of ergosterol and 9(11)-dehydroergosterol, an ergosterol derivative that has another double bond. As a result, ergosterol and 9(11)-dehydroergosterol had only minor effects on the response of the cells to LPS stimulation (Fig. 2A–D), indicating that a C-14 double bond might be the modification of ergosterol responsible for inducing tolerogenic DCs.

The potential activity of other anti-inflammatory agents with a conjugated double bond, such as conjugated linoleic acids, were also tested in comparison to 14-DHE. However, two well-known anti-inflammatory compounds, linoleic acid and conjugated linoleic acid, showed only minor ability to suppress the expression of I-A/I-E and CD86 and the production of IL-12p40 (Fig. 2A–C).

Taken together, these findings show that 14-DHE possesses unique and novel activity to suppress the expression of I-A/I-E and CD86 and the production of IL-12p40 in DCs, but it increase the production of IL-10.

### Treatment of mDCs with 14-DHE induced Tregs

**Figure 3:**
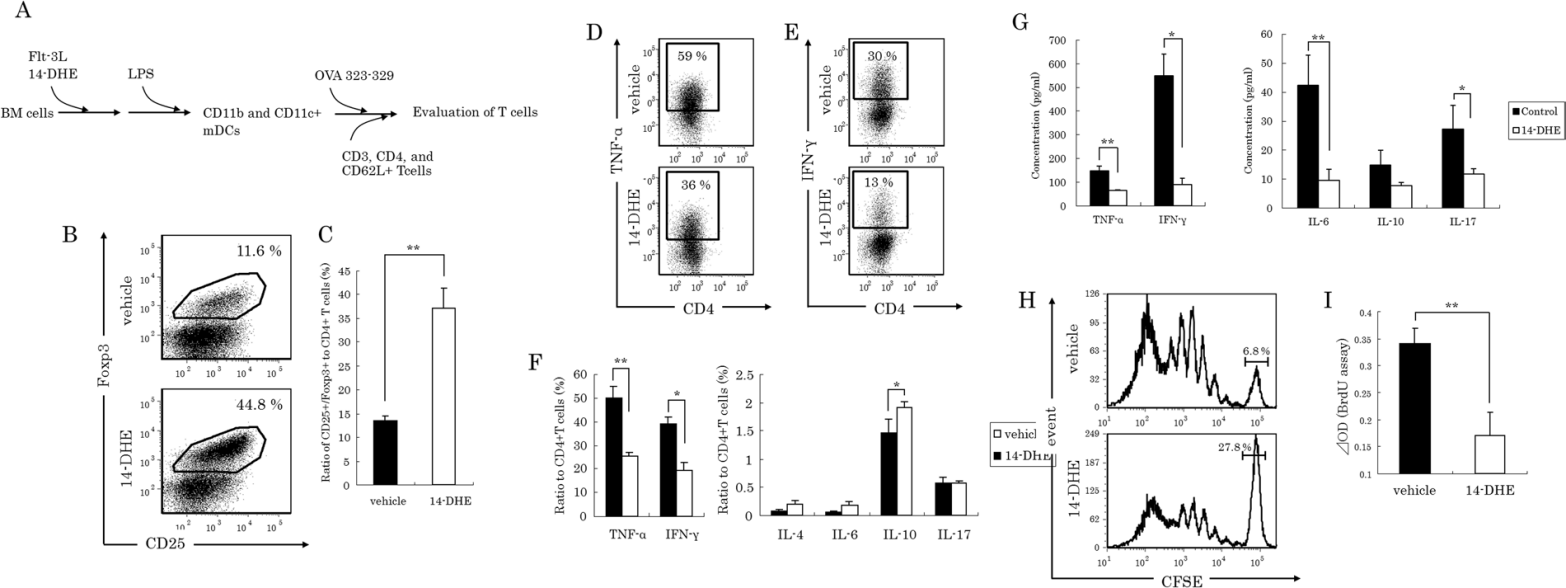
Ability of myeloid DCs treated with 14-DHE to induce naïve T cells into Treg cells (antigen-specific reactions). (**A**–**C**) CD4- and CD62L-positive T cells were purified from the spleen of OT-2 mice and cultured with 14-DHE-treated CD11b and CD11c-positive myeloid DCs schemed in (**A**). Treg cells were gated as CD4-, CD25- and Foxp3-positive cells. (**B**) Representative data of Tregs after co-culture of naïve CD4 positive T cell with mDCs treated with or without 14-DHE. (**C**) Ratio of CD25- and Foxp3-positive to CD4-positive cells gated as shown in (**B**). (**D**–**F**) After culture with mDCs, T cells were treated with Leukocyte Activation Cocktail plus BD GolgiPlug for 4 h, and the ratios of T cells producing each cytokine were analyzed by flow cytometry. CD4-positive T cells producing TNF-α (**D**) and IFN-γ (**E**) were gated, and the ratios of cells producing TNF-α, IFN-γ, IL-4, IL-6, IL-10 and IL-17 were measured (**F**). (**G**) Cytokines in the co-culture supernatant were quantified by ELISA. (**H**) Naïve T cells labeled with CFSE were cultured with mDCs, and cell division was analyzed by flow cytometry. (**I**) Cell expansion in co-culture was determined by uptake of BrdU. Data are means ± SEM of 3 wells per sample. *p < 0.05 and **p < 0.01.

Because 14-DHE-treated mDCs displayed a tolerogenic phenotype, 14-DHE-treated DCs were co-cultured with naïve CD4- and CD62L-positive T cells from OT-II TCR transgenic mice to determine whether 14-DHE has an effect on Treg induction schemed in Fig. 3A. After 5 days in culture, expansion of the T cells was observed under a microscope, and differentiation of the T cells was analyzed by flow cytometry. Tregs were gated as CD3e, CD4, CD25 and Foxp3-positive cells (Fig. 3B). The ratio of Tregs in the co-culture with 14-DHE-treated mDCs was nearly three times higher than that in the co-culture with untreated control mDCs (Fig. 3B,C). On the other hand, the ratio of TNF-α- and IFN-γ-producing CD4 T cells in the co-culture with 14-DHE-treated mDCs was significantly lower than that in the co-culture with control mDCs (Fig. 3D–F). The ratio of IL-10-producing CD4 T cells was significantly higher in the co-culture with 14-DHE-treated mDCs than in the co-culture with control mDCs (Fig. 3F). By contrast, the ratios of IL-4-, IL-6- and IL-17-producing T cells were not changed.

The cytokines in the supernatant of the co-cultures were also quantified. The amount of TNF-α and IFN-γ was also significantly lower in the supernatant of the co-culture with 14-DHE-treated mDCs than in the control co-culture, in agreement with the evaluation of intracellular cytokine-producing cells. In addition, the amount of IL-6 and IL-17 was significantly lower in the supernatant of the co-culture with 14-DHE-treated mDCs (Fig. 3G). Cellular proliferation of naïve CD4 T cells during co-culture with untreated and 14-DHE-treated mDCs was measured by CFSE fluorescence and BrdU uptake. Flow cytometry analysis using CFSE-labeled T cells showed that the cell division of T cells co-cultured with 14-DHE-treated mDCs was suppressed as compared with that of T cells co-cultured with control mDCs (Fig. 3H). BrdU uptake was also significantly reduced in the T cells cultured with 14-DHE-treated mDCs (Fig. 3I).

**Figure 4:**
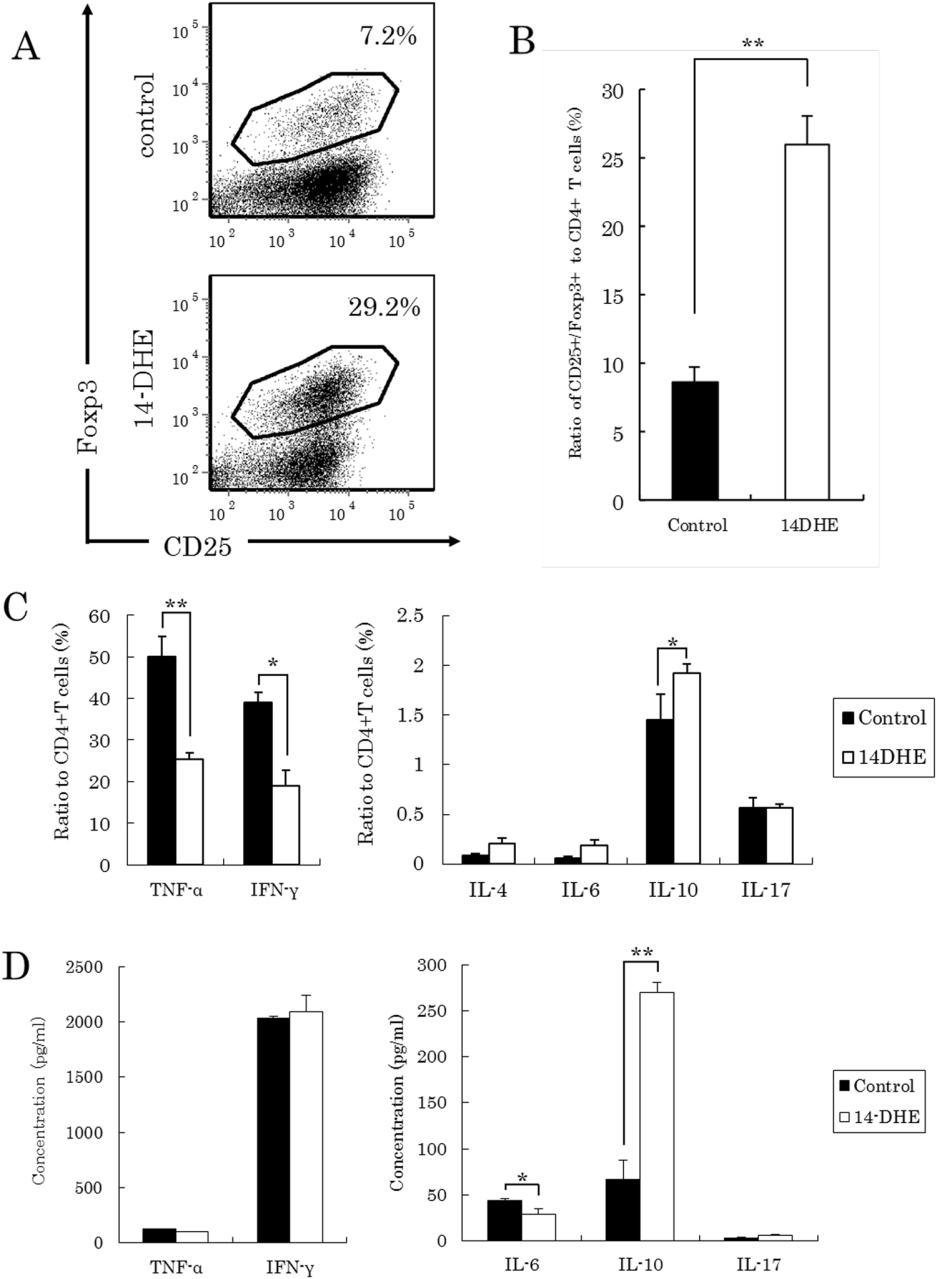
Mixed lymphoid reaction experiment using 14-DHE-treated mDCs. (**A** and **B**) CD4- and CD62L-positive T cells were purified from the spleen of BALB/c mice and cultured with 14-DHE-treated mDCs. Tregs were gated as CD4-, CD25- and Foxp3-positive cells. (**A**) Representative data of Tregs after co-culture of naïve CD4 T cells with mDC treated with or without 14-DHE. (**C**) After culture with mDCs, T cells were treated with Leukocyte Activation Cocktail plus BD GolgiPlug for 4 h, and the ratios of T cells producing each cytokine were analyzed by flow cytometry. CD4 T cells producing TNF-α, IFN-γ, IL-4, IL-6, IL-10 and IL-17 were measured. (**D**) Cytokines in the co-culture supernatant were quantified by ELISA. Data are means ± SEM of 3 wells per sample. *p < 0.05 and **p < 0.01.

Next, a series of experiments based on the previous co-culture of naïve T cells with BM-derived DCs was carried out using a co-culture of naïve T cells from BALB/c mice with BM-derived DCs from C57BL/6 mice to evaluate the effect on the differentiation of T cells. The ratio of Tregs in the co-culture with 14-DHE-treated mDCs was nearly 3 times higher than that in the co-culture with control mDCs (Fig. 4A,B) and the ratio of TNF-α- and IFN-γ-positive CD4 T cells cultured with 14-DHE-treated mDCs was also significantly lower than that in the co-culture with control mDCs (Fig. 4C). The ratio of IL-10-positive CD4 T cells in the co-culture with 14-DHE-treated mDCs was significantly higher than that in the co-culture with control mDCs. In addition, the amount of TNF-α and IL-6 in the supernatant of the co-cultures with 14-DHE-treated mDCs was also significantly reduced (Fig. 4D). These experimental data from mixed lymph reactions were essentially same as those from antigen-specific reactions. Taken together, these findings indicate that mDCs conditioned with 14-DHE have the ability to induce Treg cells from naïve CD4 T cells, and suppress inflammatory differentiation and expansion in various inflammatory situations.

### 14-DHE suppressed the development of EAE

**Figure 5:**
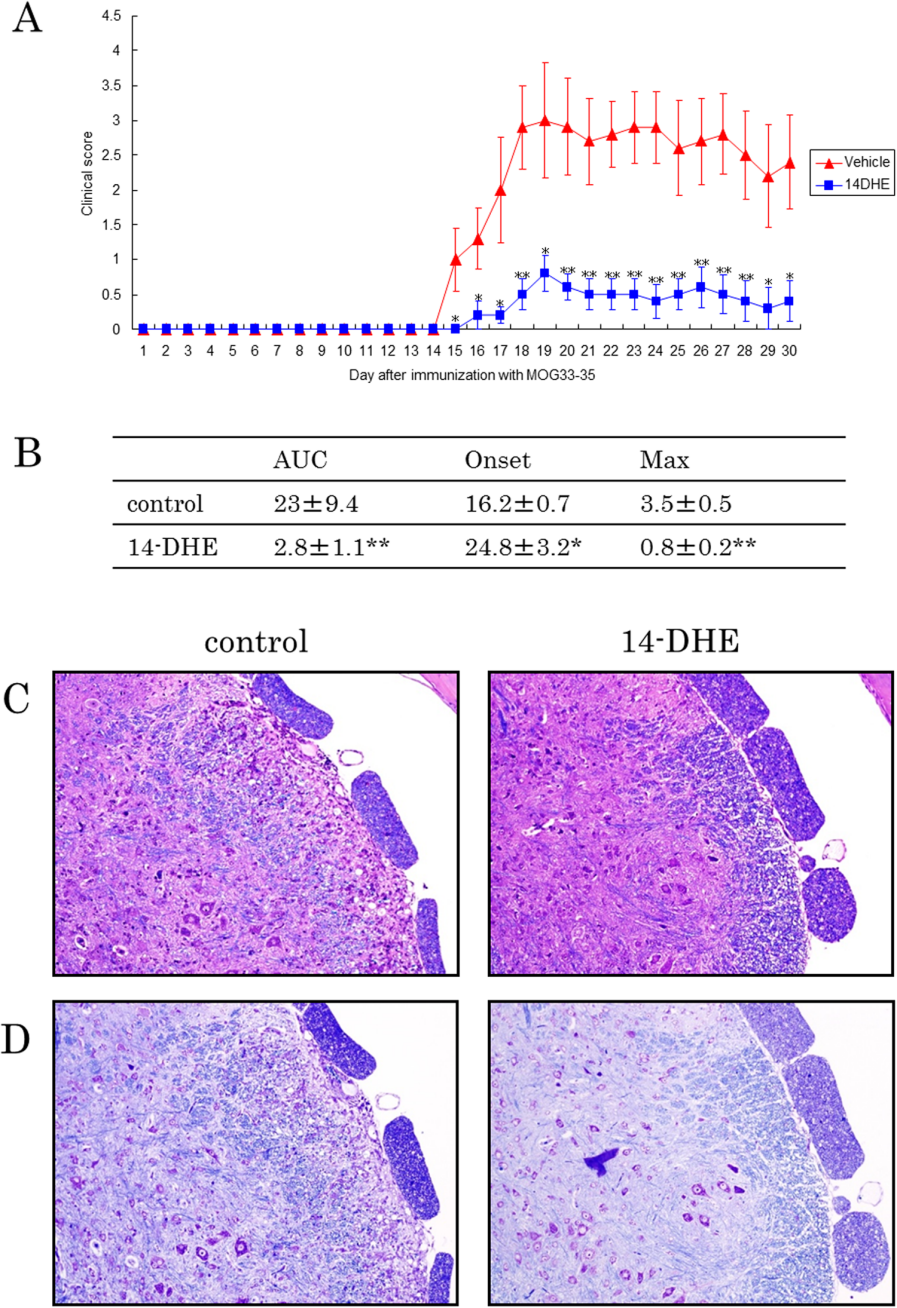
Protective effects of 14-DHE in a mouse model of experimental autoimmune encephalomyelitis. EAE was induced by immunization with MOG/CFA conjugated with PTx. Mice were simultaneously immunized subcutaneously in one flank on day 0 and in the other on day 7 with 0 or 0.4 mg/kg 14-DHE in a different location from the MOG injection. (**A**) Clinical score of diseased mice. (**B**) Clinical features of total clinical score points (AUC), day of disease onset, and maximum score of disease. (**C**) Hematoxylin and eosin stain of spinal cords on day 30 after immunization. (**D**) Klüver-Barrera stain of spinal cords on day 30 after immunization. Data are means ± SEM of 5 mice per group. *p < 0.05 and **p < 0.01.

To determine whether 14-DHE has a beneficial effect on inflammatory disease *in vivo*, it was tested in the murine model of EAE. Each mouse was subcutaneously injected with 0.4 mg/kg of 14-DHE and, in a different location, 8 mg/kg of myelin oligodendrocyte glycoprotein peptide 35–55 (MOG) as an antigen on days 0 and 7. Scoring showed that the severity of disease in the 14-DHE group was significantly lower than that in the control group as on day 15 (Fig. 5A). After day 15, the score in the group of 14-DHE treatment remained lower than that of the control group. In detail, the total score (area under the curve [AUC]), disease onset date, and maximum score were calculated from day 0 to day 30 after immunization with the MOG peptide (Fig. 5B). The AUC was 8 times lower, the onset date was 8 days later, and the maximum score was 4 times lower in the 14-DHE group than in the control group. In addition, histopathological analysis (hematoxylin-eosin staining and Klüver-Barrera staining) showed that loss of myelin sheath and infiltration of lymphocytes were suppressed in the 14-DHE treated group (Fig. 5C and D, respectively). Mice treated with 14-DHE did not develop EAE disease. To check for the possibility of a direct interaction between 14-DHE and MOG peptide, we demonstrated that intraperitoneal injection of 14-DHE and the subcutaneous injection of 14-DHE in an injection site different to that of the MOG peptide also suppressed the development of EAE disease (data not shown).

### 14-DHE suppressed inflammation and induced Tregs in response to EAE induction

**Figure 6:**
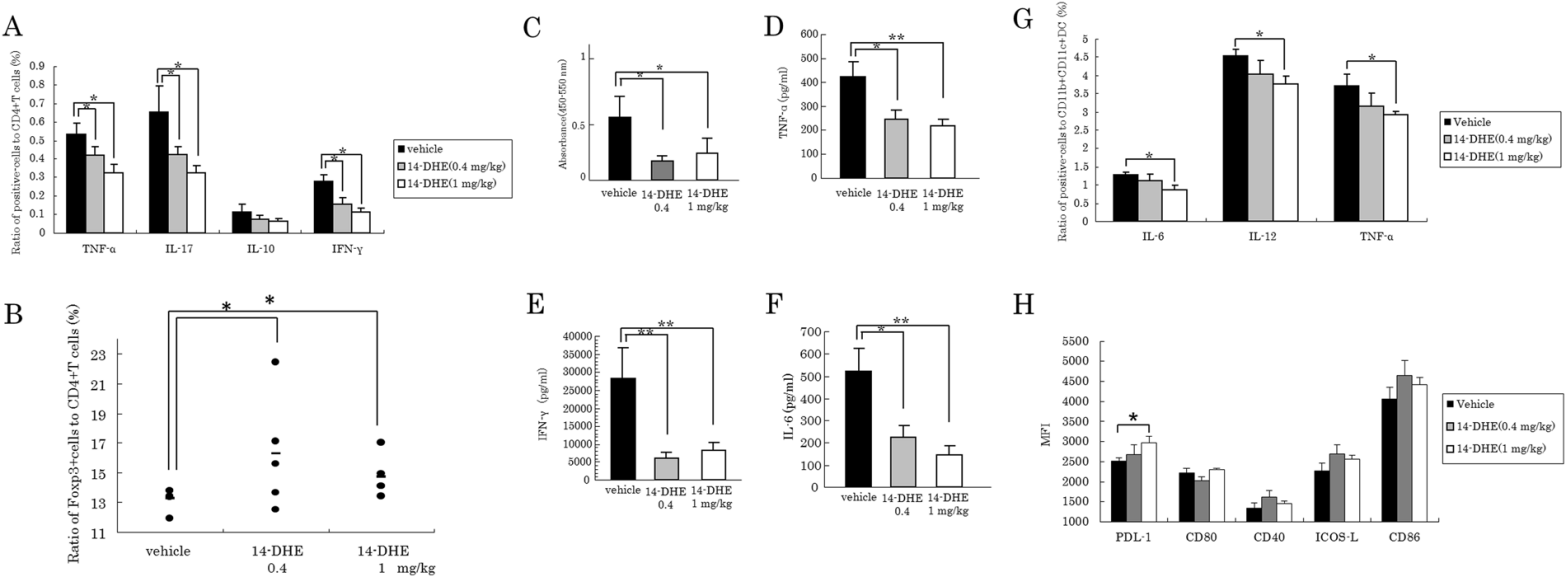
14-DHE suppressed the production of inflammatory cytokines and cell expansion, and increased Tregs in LN T cells. (**A** and **B**) To detect cytokine-producing cells and Tregs, LN cells from mice immunized with MOG/CFA were cultured under stimulation with Leukocyte Activation Cocktail plus BD GolgiPlug, and analyzed by flow cytometry. (**A**) Ratios of TNF-α-, IL-17-, IL-10- and IFN-γ-producing cells among CD3e and CD4-positive cells. (**B**) Ratios of CD3e, CD4, CD25 and Foxp3-positive cells among CD3e and CD4-positive cells. (**C**–**F**) To detect cell proliferation and cytokine production, LN cells were cultured with 2 μM MOG. BrdU was added to the cell culture to detect cell proliferation. (**C**) Concentrations of pro-inflammatory cytokines produced in response to re-stimulation with MOG antigen. (**D**,**E**, and **F**) ELISA quantification of TNF-α, IFN-γ, and IL-6, respectively, in the cell supernatant. (**G**) To detect cytokine production in mDCs, LN cells were stimulated with Leukocyte Activation Cocktail plus BD GolgiPlug, and the ratio of IL-6-, IL-12p70-, and TNF-α positive cells was measured among CD11b and CD11c-positive cells by flow cytometry. (**H**) Expression of the cell-surface markers PDL-1, CD80, CD40, ICOS-L and CD86 on CD11b and CD11c-positive myeloid DCs was analyzed by flow cytometry.

The intracellular cytokine production of CD4 T cells distributed in inguinal and axilla LNs from mice on day 11 after first immunization was measured by flow cytometry. The ratio of TNF-α-, IL-17-, and IFN-γ-producing CD4 T cells was significantly lower in a dose-dependent manner in the 14-DHE treated group than in the control group (Fig. 6A). Flow cytometry analysis of Treg cells, gated as CD3e, CD4, CD25 and Foxp3-positive cells, in the LNs also showed that the ratio was significantly higher in the 14-DHE treated group than in the control group (Fig. 6B). The expansion of lymphocytes in the LNs due to MOG peptide stimulation was also suppressed in the 14-DHE treated group (Fig. 6C). 14-DHE suppressed the production of TNF-α (Fig. 6D), IFN-γ (Fig. 6E) and IL-6 (Fig. 6F) in LN cells.

To determine the phenotype of myeloid DCs in LNs, CD11b and CD11c-positive cells were analyzed by flow cytometry. The ratio of IL-6-, IL-12p40/p70-producing cells was significantly lower (Fig. 6G), whereas the expression of PDL-1 on mDCs in the LNs was significantly increased (Fig. 6H) in the 14-DHE treated group. Together, these results revealed that 14-DHE suppressed mDC activation and inflammatory responses of T cells and induced Tregs *in vivo*.

### 14-DHE suppressed the inflammatory phenotype in DCs

**Figure 7:**
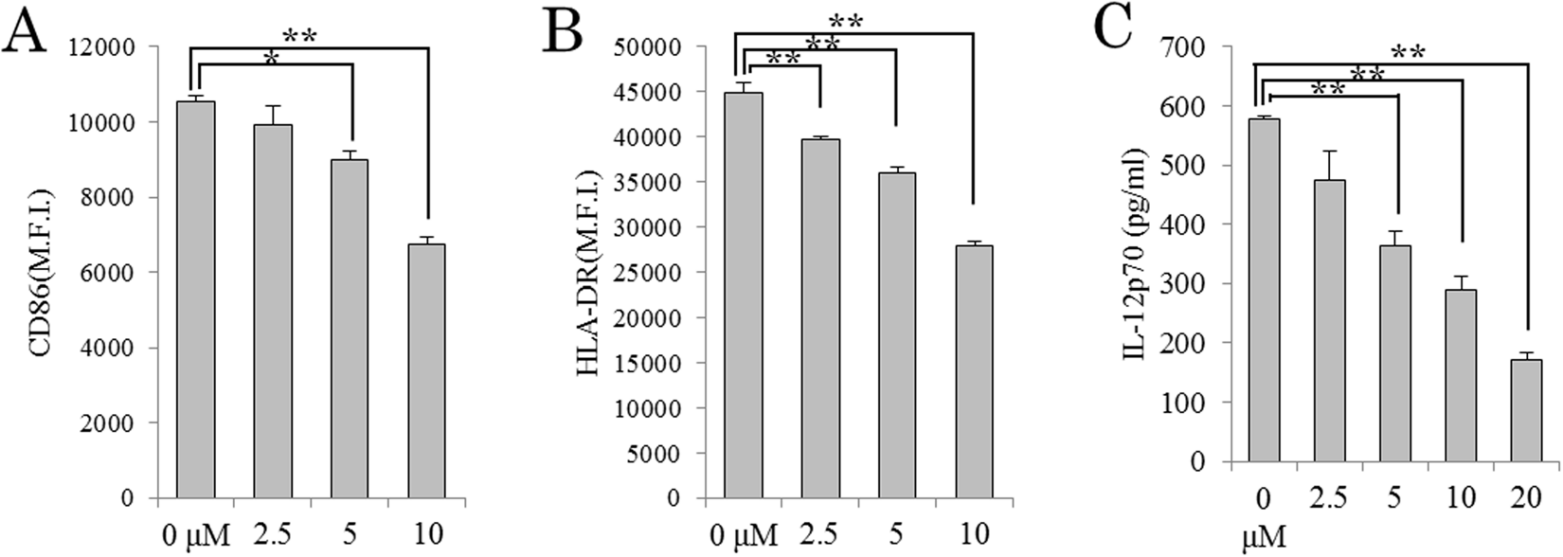
Effects of 14-DHE on human DCs inducing tolerogenic phenotype. CD14-positive cells in human PBMCs were cultured in medium containing 50 ng/ml of GM-CSF and 20 ng/ml of IL-4 in the presence of 14-DHE, and treated with 1 μg/ml of LPS and 100 ng/ml of IFN-γ on the sixth day of culture. (**A** and **B**) Expression of CD86 (**A**) and HLA-DR (**B**) in CD11c-positive cells was analyzed by flow cytometry. (**C**) IL-12p70 in the culture supernatant was quantified by ELISA. Data are means ± SEM of 3 wells per sample. *p < 0.05 and **p < 0.01.

To determine the effects of 14-DHE on human DCs, cells were induced from human PBMCs with GM-CSF and IL-4. In CD11c-positive DCs treatment with 14-DHE suppressed the expression CD86 (Fig. 7A) and HLA-DR (Fig. 7B) in response to LPS and IFN-γ stimulation. Production of IL-12p70 was also significantly suppressed by treatment with 14-DHE (Fig. 7C). From these data, 14-DHE was demonstrated to be effective on human as well as murine DCs.

## Discussion

In the present study, we have identified 14-DHE as a novel anti-inflammatory agent in the Japanese traditional fermented food material Koji by monitoring the induction of tolerogenic DCs. 14-DHE was first identified in 1951, but its biological function was not elucidated^25^. Although there have been many studies regarding the improvement of food flavor and taste with Koji^24^, few findings on its biological effects have been reported. Here, ergosterol and 9(11)-dehydroergosterol showed only minor effects of anti-inflammation; however, 14-DHE showed a much higher activity to suppress both the production of IL-12 and the expression of MHC class II and co-stimulatory molecules such as CD86. Furthermore, 14-DHE also showed much higher activity than linoleic acids and conjugated linoleic acids, which are known anti-inflammatory compounds derived from food materials^26–29^.

14-DHE suppressed the expression of activation and/or pro-inflammatory markers of DCs such as CD86, CD80, and MHC classII against LPS stimulation without affecting the expression of ICOS-L, an anti-inflammatory marker of DCs. 14-DHE also suppressed the production of IL-12, but not IL-10, in response to LPS stimulation. 14-DHE-treated DCs also showed a tolerogenic phenotype in response to other TLR ligands, such as CpG and Pam3 (data not shown). CD86 and CD80 are reported to work as co-stimulatory factors that induce T cells into inflammatory phenotypes, such as Th1 and Th17, and ICOS-L and PDL-1 are known to be involved in anti-inflammatory T cell phenotypes, such as Tregs^30–32^. These results suggest that 14-DHE-treated DCs induce naïve T cells into an anti-inflammatory or regulatory type. Furthermore, we demonstrated that these effects also occur in human cells.

The capability of 14-DHE-treated DCs to induce CD25- and Foxp3-positive Tregs was confirmed as a result of co-culture with naïve T cells. In addition, the expansion of T cells was suppressed and the ratios of pro-inflammatory T cells, such as IFN-γ- and IL-17-producing cells, were reduced. We confirmed that the direct treatment of naïve T cells with 14-DHE alone did not induce into Tregs and 14-DHE did not have an effect on the proliferation of induced Treg (data not shown), and that 14-DHE affected Treg induction via modification of DC phenotype. It has previously been reported that DCs treated with 50 μM curcumin contained in turmeric powder show to induce naïve T cells into Tregs^33^. Relative to curcumin, 14-DHE might show similar activity at a lower concentration (3 μM). 14-DHE also showed potent activity as compared with previously reported conjugated linoleic acids^34^ that is, DCs treated with 50 μM conjugated linoleic acids displayed the same phenotype as those treated with 3 μM 14-DHE. Thus, the activity of 14-DHE is potent relative to previously reported food components.

To evaluate the capability of 14-DHE to induce tolerogenic DCs *in vivo*, 14-DHE was administered the mouse model of EAE. Subcutaneous administration of 14-DHE suppressed the clinical scores, infiltration of neutrophils and demyelination. Intraperitoneal administration also suppressed the EAE clinical score. Ratios of T cells producing IL-17, IFN-γ, and TNF-α were significantly reduced, and that of Tregs was significantly increased in infra-axillary and inguinal LNs in EAE model mice. The ratio of mDCs producing IL-12 was significantly decreased and the expression of PDL-1 on mDCs was significantly increased. As noted above, 14-DHE did not directly cause T cells to differentiate into Tregs; therefore, these findings suggest that 14-DHE modifies DCs into tolerogenic DCs *in vivo*, and these tolerogenic DCs then induce Tregs, leading to a suppression of inflammatory responses and clinical scores. Further studies are required to evaluate the effects of oral administration of 14-DHE on DCs *in vivo*. In the further study, the effects of 14-DHE orally administered on inflammatory model mice should be examined.

Japanese foods are regarded as healthy foods, and the present study has elucidated a new aspect of evidence supporting these health claims. 14-DHE might be a helpful aid in the prevention of chronic inflammatory diseases and autoimmune diseases.

## Methods

### Mice

Six-week-old C57BL/6 J and BALB/c female mice were purchased from Charles River Laboratory in Kanagawa, Japan. OT-II CD4-Tg mice specific for OVA peptide 323–339 were purchased from Jackson Laboratories (ME, USA). All animals were maintained in a pathogen-free facility. All experiments were performed in accordance with the guidelines for the care and use of laboratory animals of Kirin Company Ltd. The studies were approved by the Committee for Animal Experimentation Kirin Company (approval numbers: YO11-00027, YO11-00072, YO11-00198, YO12-00180). All efforts were made to minimize animal suffering.

### Antibodies

Anti-CD11b-APC-Cy7 (M1/70), anti-CD25-APC-Cy7 (PC61), anti-IL4-APC, anti-IL-12p40/p70-APC, and anti-IL-17-PE antibodies were purchased from BD Pharmingen (CA, USA). Anti-B220-PerCP (RA3-6B2), anti-CD4-APC (L3T4), anti-CD11c-PE-Cy7 (N418), anti-I-A/I-E-FITC (M5/114.15.2), anti-CD80-APC (16-10A1) anti-CD86-APC (GL1), anti-Foxp3-PE-Cy7 (FJK-16a), anti-IL-6-PE (MP5-20F3), anti-IFN-γ-PE-Cy7 (XMG1.2), anti-TNF-α-FITC (MP6-XT22), and anti-IL-10-APC (JES5-16E3) antibodies were purchased from eBioscience (CA, USA).

### Isolation and identification of 14-DHE

To prepare the wheat bran mold starters, *Aspergillus awamori* (mild ko-ji, Akita Konno CO. Ltd., Akita, Japan) was inoculated for 3 days into wheat bran soaked in water and steamed. The mold starters (30 g) were freeze-dried and crushed, and then extracted using a sonicator (Ultrasonic Cleaner, Shibata, Japan) in 100 mL of ethanol. After evaporation of the solvent, the ethanol extract was partitioned between methanol and hexane. Solid-phase extraction of the hexane phase was achieved by using a silica column (Bond Elut-SI, Varian, CA, USA) with a hexane-ethyl acetate gradient. Final purification was obtained by HPLC (Shimadzu, Kyoto, Japan) using a UK-silica column (Unison, 10 mm × 250 mm, Imtakt, Kyoto, Japan) with a hexane-isopropanol (98:2) gradient, followed by a C30-UG-5 column (Develosil, 10 mm × 250 mm, Nomura Chemical Co. LTD., Aichi, Japan) with an acetonitrile-isopropanol (99:1) gradient. Compounds were detected at an absorbance wavelength of 320 nm.

### Structural analysis

The molecular formula of compound **1** was determined to be C_28_H_42_O (ergosterol - 2 H) by HRESI-MS analysis (*m/z* 395.33088 (M + H)^+^, calcd. for C_28_H_43_O, 395.33139). Because the ^1^H and ^13^C NMR spectra of compound **1** were closely related to those of ergosterol, compound **1** was strongly suggested to possess a dehydro (2 H) ergosterol structure. The characteristic UV spectrum of compound **1** (λmax 321 nm in MeOH) indicated that compound **1** possessed a triene structure composed by Δ^5,6^, Δ^7,8^, and a double bond conjugated to Δ^5,6^ or Δ^7,8^. The position of the new double bond was analyzed via the 2D NMR spectra (^1^H-^1^H DQF COSY, HMQC, and HMBC), and compound **1** was determined to be 14-dehydro ergosterol (14-DHE).

^1^H NMR (CDCl_3_) δ: 0.83 (d, *J* = 6.3 Hz, 3 H, H-26), 0.85 (d, *J* = 8.5 Hz, 3 H, H-27), 0.89 (s, 3 H, H-18), 0.92 (s, 3 H, H-19), 0.93 (d, *J* = 7.3 Hz, 3 H, H-28), 1.05 (d, *J* = 6.8 Hz, 3 H, H-21), 1.45 (2 H, H-2 and H-25), 1.55–1.60 (2 H, H-12 and H-17), 1.71 (m, 1 H, H-11), 1.84–1.90 (4 H, H-1, H-2, H-16, and H-24), 2.02–2.07 (2 H, H-9 and H-12), 2.18–2.22 (2 H, H-16 and H-20), 2.30 (m, 1 H, H-4), 2.51 (ddd, *J* = 2.2, 5.1, 10.6 Hz, H-4), 3.64 (m, 1 H, H-3), 5.21 (dd, *J* = 7.9, 15.1 Hz, 1 H, H-22), 5.27 (dd, *J* = 7.0, 15.1 Hz, 1 H, H-23), 5.65 (dd, *J* = 2.2, 5.9 Hz, 1 H, H-6), 6.15 (m, 1 H, H-7).

^13^C NMR (CDCl_3_) δ: 14.5 (C-18), 16.8 (C-19), 17.6 (C-28), 19.6 (C-26), 19.7 (C-27), 19.9 (C-11), 21.1 (C-21), 32.0 (C-2), 33.1 (C-25), 36.0 (C-16), 37.0 (C-10), 37.8 (C-1), 38.9 (C-12), 39.0 (C-20), 41.0 (C-4), 42.8 (C-24), 45.4 (C-13), 46.3 (C-9), 58.1 (C-17), 70.4 (C-3), 117.4 (C-7), 120.4 (C-15), 120.5 (C-6), 132.0 (C-8), 132.2 (C-23), 135.4 (C-22), 143.0 (C-5), 149.2 (C-14).

### Generation of BM-derived DCs

BM cells were isolated from C57BL/6 J female mice by conventional methods^35^ and suspended at 1.0 × 10^6^ cells/mL in DC culture medium composed of complete RPMI 1640, 10% heat-inactivated FCS (Invitrogen, CA, USA), 2.5 mM 4-(2-hydroxyethyl)-1-piperazineethanesulfonic acid (HEPES) buffer (Invitrogen), 1 mM sodium pyruvate (Invitrogen), 50 μM 2-mercaptoethanol (Invitrogen), 1% non-essential amino acids (NEAA, Invitrogen), 100 U/mL of penicillin, and 100 μg/mL of streptomycin (Sigma, MO, USA). The cells were cultured in the presence of 100 ng/mL of Flt-3L (R&D systems, MN, USA) and test extracts, fractions, and compounds were maintained at 37 °C in a 5% CO_2_ atmosphere. In some assays, BM cells were treated with sample on day 6. On day 7, the cells were stimulated with 5 ng/mL of lipopolysaccharide (LPS) (Sigma). After overnight stimulation, the cells were treated with antibodies, and the expression of cell surface markers was evaluated by flow cytometry using a FACSCanto II instrument BD Biosciences, CA, USA). The production of cytokines IL-12p40 and IL-10 in the supernatant of cell cultures after LPS stimulation was measured by using ELISA kits (BD Pharmingen). In the phase of screening step, each sample was evaluated repeatedly in the two wells, and after identification of 14-DHE, samples at each concentration were evaluated in the three wells.

### *In vitro* stimulation of T cells

Naïve CD4 T cells from the spleens of BALB/c and OT-II mice were isolated with the CD4^+^CD62L^+^ T Cell Isolation Kit II (Miltenyi Biotec, Auburn, CA). For the cell division assay, naïve CD4 T cells were labeled with carboxyfluorescein diacetate succinimidyl ester (CFSE) (Invitrogen) following the manufacturer’s instructions. BM-derived mDCs (gated as CD11b^+^CD11c^+^ cells) were induced with Flt-3L, treated on day 0 with 0 or 3 μM 14-DHE, stimulated on day 7 with 5 ng/mL of LPS, and then sorted with FACSAria (BD Biosciences). Sorted mDCs were treated with 0.1 μM OVA 323–329 for 30 min, followed by 10 μg/mL of mitomycin C for 30 min at 37 °C. mDCs and T cells were co-cultured in DC culture medium containing 0.1 μM OVA 323–329 and 0.5 ng/mL of recombinant TGF-β (R&D systems). For mixed lymphoid reactions, T cells from BALB/c (4.0 × 10^5^ cells) and mDCs from C57BL/6 J (8.0 × 10^4^ cells) were cultured at a ratio of 5:1 for 5 days. For antigen-specific reactions, T cells from OT-II (4.0 × 10^5^ cells) and mDCs from C57BL/6 J (1.6 × 10^4^ cells) were cultured at a ratio of 25:1 for 5 days. Before proceeding the assay, the adequate ratio of co-culture to evaluate the effect of 14-DHE was examined.

To measure intracellular cytokines, cells were stimulated for 4 h with Leukocyte Activation Cocktail plus BD GolgiPlug (BD Biosciences) and treated with a BD Cytofix/Cytoperm Fixation/Permeabilization Kit (BD Biosciences) and antibodies. To detect Tregs, cells were treated with a FoxP3 Staining Kit (BD Biosciences) and antibodies. Cells producing specific cytokines and Treg cells were analyzed by flow cytometry. Cell proliferation was measured by using a flow cytometer to detect CFSE-positive cells and also by a bromodeoxyuridine (BrdU)-Cell Proliferation Assay kit (Calbiochem, Merck Group, Darmstadt, Germany).

### Induction and clinical evaluation of experimental autoimmune encephalomyelitis

C57BL/6 J mice (n = 10 mice per group for scoring; n = 5 mice per group for histopathological analysis and biochemical analysis) were immunized subcutaneously in one flank on day 0 and in the other flank on day 7 with 8 mg/kg of myelin oligodendrocyte glycoprotein peptide 35–55 (MOG) (MEVGWYRSPFSRVVHLYRNGK), which was synthesized and purified by HPLC (Operon Biotechnologies, Tokyo, Japan) and emulsified in CFA (1:1), which consists of IFA containing 5 mg/mL of *Mycobacterium tuberculosis* H37RA (Difco Laboratories, BD Biosciences) as previously described^36,37^. Each mouse was also simultaneously immunized subcutaneously in one flank on day 0 and in the other flank on day 7 with 0, 0.4, or 1.0 mg/kg 14-DHE at a location differing from the MOG injection. Pertussis toxin (8 μg/kg) was injected intravenously on days 1 and 3. After the first immunization, the severity of experimental autoimmune encephalomyelitis (EAE) was monitored and graded on a scale of 0–5 as follows: 1, limp tail; 2, hind limb weakness; 3, hind limb paralysis; 4, hind and fore limb paralysis; 5, morbidity and death.

For histological analysis, spines were harvested and fixed with neutral 10% formalin 30 days after the first immunization. Spinal cords were then extracted and embedded in paraffin. Sections were stained with hematoxylin and eosin. For biochemical analysis of lymphocytes in lymph nodes (LNs) and spleen by flow cytometry and ELISA, lymphocytes from the inguinal and axilla LNs and spleen of mice were collected 11 days after the first immunization via conventional methods^37^. In brief, removed tissues were minced in Mg^2+^ and Ca^2+^-free Hank’s Balanced Salt Solution and digested with 1 mg/mL of collagenase (Sigma) and 0.2 mg/mL of DNase I for 20 min at 37 °C. EDTA was added to 30 mM, and the mixture was incubated for 10 min at room temperature. Tissue lysates were filtered through a 70-μm nylon cell strainer. The collected LN cells and splenic lymphocytes were cultured for 7 days in the absence or presence of 2 μM MOG. Cytokines in the cell culture supernatants were detected by using ELISA kits for IFN-γ (BD Biosciences), IL-17 (R&D systems), and TNF-α (eBioscience). Intracellular cytokines, Treg cells, and BrdU cell proliferation were detected by previously described methods. For the evaluation of monocytes, the collected lymphocytes were layered onto 15% Histodenz (Sigma) in RPMI 1640 containing 10% FCS and centrifuged at 450 x g for 20 min without braking. The low-density fractions at interfaces were collected, washed, and analyzed by flow cytometry.

### Generation of human PBMC-derived DCs

Human peripheral blood mononuclear cells (PBMCs) from healthy, HIV, HBV, HCV and HTLV- donors were purchased from Lonza Japan (Tokyo, Japan). CD14-positive cells were isolated from human PBMCs with CD14 microbeads (Miltenyi Biotec) and cultured in the RPMI 1640 medium containing 10% FBS (Invitrogen), 50 U/ml of penicillin and 50 μg/ml of streptomycin (Invitrogen), 50 μmol/l of 2- mercaptoethanol (Invitrogen), 50 ng/ml of GM-CSF (R&D systems), and 20 of ng/ml IL-4 (R&D systems). Cells were cultured in the presence of 14-DHE at a concentration of 0, 2.5, 5, 10, and 20 μM. On 6 day of culture, cells were treated with 1 μg/ml of LPS (Sigma) and 100 ng/ml of IFN-γ (Sigma). After 24 hours, the supernatant was collected for the quantification of IL-12p70 (BD Pharmingen), and cells were collected for analysis by flow cytometry. Cells were stained with anti-CD86-PE (IT2.2, eBiosciences), anti-CD11c-PE-Cy7 (B-ly6, BD Pharmingen), anti-HLA-DR-PerCP (L243, BD Bioscience) and 7AAD (BD Bioscience), and the expression of CD86 and I-A/I-E in CD11c-positive and 7AAD-negative was assessed cells. This experiment was in accordance with the guidelines of Kirin Company Ltd and approved by the clinical research ethics committee of Kirin Company (approved number: 11–01).

### Statistical analysis

All values are expressed as means ± SEM. Data from the cytokine production assays and flow cytometry analysis *in vitro* and *in vivo* were analyzed by one-way ANOVA, followed by Tukey-Kramer’s test or Student’s t-test. All statistical analyses were performed by using the Ekuseru-Toukei 2012 software program (Social Survey Research Information, Tokyo, Japan).

### Data availability statement

The datasets generated during and/or analyzed during the current study are available from the corresponding author on reasonable request.
